# Radiation-Induced Degradation of a Cold-Redundant DC/DC Converter Under Total Ionizing Dose Stress

**DOI:** 10.3390/mi17020197

**Published:** 2026-01-31

**Authors:** Xiaojin Lu, Zhujun Xi, Qifeng He, Ziyu Zhou, Mengyao Li, Liangyu Xia, Gang Dong

**Affiliations:** 1 Faculty of Integrated Circuit, Xidian University, Xi’an 710071, China; 2 The 43rd Research Institute of China Electronics Technology Group Corporation, Hefei 230088, China; 3 Shanghai Institute of Space Propulsion, Shanghai 201112, China; 4 Shanghai Institute of Organic Chemistry, Chinese Academy of Sciences, Shanghai 201112, China; 5 School of Electrical and Electronic Engineering, Huazhong University of Science and Technology, Wuhan 430074, China

**Keywords:** DC/DC converter, cold redundancy, total ionizing dose (TID), VDMOS

## Abstract

This paper investigates the degradation characteristics of a DC/DC converter operating under cold redundancy conditions when subjected to total ionizing dose (TID) effects. An optimized RCC isolated auxiliary power supply circuit was evaluated through ^60^Co γ-ray irradiation up to 100 krad(Si) at dose rates of 3.89, 8.89, and 13.89 rad (Si)/s, with electrical characterizations performed at both the system level and the device level, focusing on the critical VDMOS transistors. The results indicate that the main output voltage and conversion efficiency remain essentially stable after irradiation, whereas the auxiliary supply voltage and efficiency degrade significantly, leading to a pronounced reduction in the controller supply margin. Device-level measurements reveal a negative threshold voltage shift of approximately 0.5–1.0 V with clear dose-rate dependence, while the subthreshold swing shows no obvious variation, suggesting that the degradation is primarily dominated by oxide-trapped charge effects. In addition, a substantial increase in drain current at low gate voltages is observed, which may further exacerbate restart risks under cold redundancy conditions. These findings demonstrate that the auxiliary power supply and startup margin constitute critical vulnerability points of cold-redundant DC/DC converters under TID stress and should therefore be primary targets for radiation-hardened design.

## 1. Introduction

The spacecraft power system is the core subsystem for energy generation, conversion, and distribution; its performance and reliability directly determine mission success and on-orbit lifetime. As a key functional unit of the power system, the DC/DC converter performs bus power conversion and distribution, provides regulated power to payloads and other subsystems, and ensures power quality [1]. Consequently, its reliability is often a decisive factor for overall spacecraft availability and operational lifetime [2]. In the space radiation environment, DC/DC converters face not only transient disturbances and catastrophic failure risks induced by single-event effects (SEE), but also the long-term accumulation of total ionizing dose (TID), which can cause parameter drifts in power devices and control/drive circuits, efficiency degradation, reduced loop margins, and even functional failure [2,3,4,5]. Prior studies have shown that power MOSFETs and their associated control paths in switching converters are sensitive to both TID and SEE, with system-level degradation commonly manifested as efficiency loss, degraded regulation capability, and abnormal startup or restart behavior [3,4]. In addition, threshold-voltage shifts and leakage-current variations in high-voltage MOSFETs can further impact converter losses and operating boundaries [6].

To meet the stringent reliability requirements of space missions, redundancy is widely adopted in spacecraft power architectures. Among various schemes, cold redundancy refers to the configuration in which one unit operates while the redundant units remain completely powered off [7,8], offering the intuitive benefit of reduced electrical stress and aging. However, in a radiation environment, “power-off” does not necessarily mean “no degradation”. TID can induce charge trapping and defect generation in oxides and at interfaces, leading to parameter shifts even under unbiased conditions; such degradation may manifest upon re-powering as insufficient startup margin or abnormal bias-supply behavior [9,10]. More importantly, experimental studies have reported that DC/DC converters irradiated in shutdown/sleep (passive-reserve) states may exhibit reduced TID tolerance and can even represent a worst-case condition, challenging the engineering intuition that cold redundancy is inherently more radiation robust [11]. Moreover, the survivability and failure thresholds after irradiation can be strongly influenced by the control chain and restart mechanism, indicating that the “cold-redundancy-restart” scenario must be explicitly evaluated [4].

It should be emphasized that studies on unpowered/sleep-mode irradiation are not absent. For example, Kessarinskiy et al. irradiated DC/DC converters in passive-reserve (unpowered/sleep) conditions and observed a pronounced reduction in TID tolerance compared with powered operation [11]. In parallel, TID-induced parametric drift and threshold evolution under standby/unbiased conditions have also been reported in other technologies; for instance, Bagatin et al. observed dose-dependent evolution of threshold-voltage distributions in 3-D NAND flash memories [12]. Nevertheless, for the engineering demands of long-duration, cold-redundant power-off operation in space missions, generalizable converter-level conclusions remain limited, particularly in two aspects: (i) systematic comparisons of multi–dose-rate behavior under unpowered irradiation and (ii) a clear system-to-device correlation linking post-irradiation startup/restart anomalies and bias-margin degradation to the degradation of key power devices, which hinders reliable failure attribution and targeted hardening design.

On the other hand, the fundamental physical mechanisms of TID effects—oxide-trapped charge buildup, radiation-induced interface-trap generation, and their time- and temperature-dependent evolution—are well established in the literature [13,14,15,16,17,18]. Threshold-voltage shifts in power MOSFETs exhibit strong dose-rate and bias dependence and may involve rebound or super-recovery behaviors, making conclusions drawn from a single dose rate or a single characterization point difficult to extrapolate to long-duration, low-dose-rate missions [10,19]. Therefore, a more closed-loop evidence chain is needed to explicitly correlate system-level anomalies under cold redundancy and multi–dose-rate irradiation (e.g., startup behavior, bias margins, and no-load current) with degradation parameters of key devices (e.g., VDMOS transistors), thereby supporting reliable failure attribution and engineering-oriented radiation hardening.

Accordingly, this work investigates the performance degradation of a DC/DC converter module operating in a cold-redundant (powered-off) state under TID stress and the underlying mechanisms. Overall, this work identifies the system-level worst-case degradation trend across multiple dose rates under cold-redundant (long-term powered-off) TID stress and establishes a closed-loop correlation between restart-relevant module signatures and critical VDMOS degradation, thereby enabling reliable failure attribution and actionable hardening guidance.

## 2. Circuit Schematic Block Diagram

**Figure 1:**
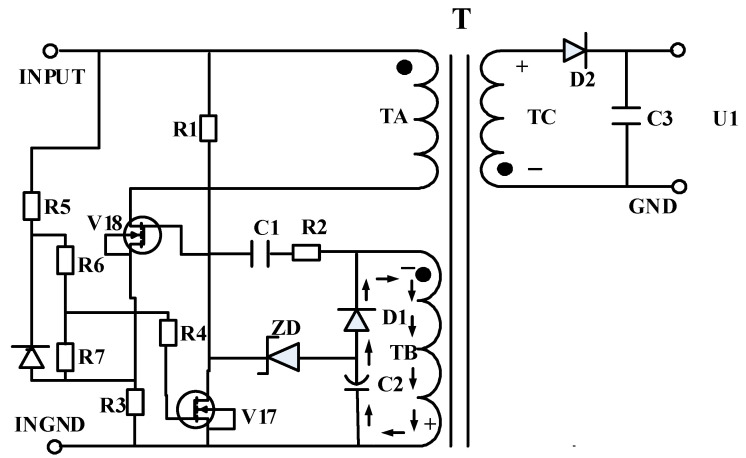
Optimized isolated auxiliary power supply schematic.

The object investigated in this work is an RCC-optimized circuit [20], whose circuit topology is shown in Figure 1. This circuit was originally designed to withstand neutron irradiation in nuclear detonation tests and is intended for future deployment in nuclear-related equipment, where it serves as a core power-conversion unit. The product has demonstrated tolerance to a neutron fluence of 3 × 10^13^ n/cm^2^; however, its tolerance to total ionizing dose (TID) effects has not yet been characterized. In particular, the TID response under cold-redundant operating conditions remains unknown.

Motivated by this gap, this study conducts TID irradiation experiments under different dose-rate conditions, with a total accumulated dose of 100 krad(Si), to systematically investigate the radiation-induced degradation behavior of the RCC-optimized circuit in the cold-redundant state.

Total ionizing dose (TID) cold-redundancy irradiation experiments were conducted on two switching VDMOS devices, denoted as V17 and V18, in the RCC auxiliary power supply circuit. In the RCC topology, V18 functions as the main power-switching transistor, while V17 serves as the auxiliary control switch; both devices share the same type and specifications. By investigating the optimized RCC circuit under cold-redundant TID irradiation at different dose rates, the performance degradation trends of the converter were systematically evaluated. Furthermore, the radiation-induced degradation mechanisms of the VDMOS devices were analyzed, and several practical hardening-oriented design measures were proposed to mitigate TID-induced performance degradation.

## 3. Experimental Conditions

**Figure 2:**
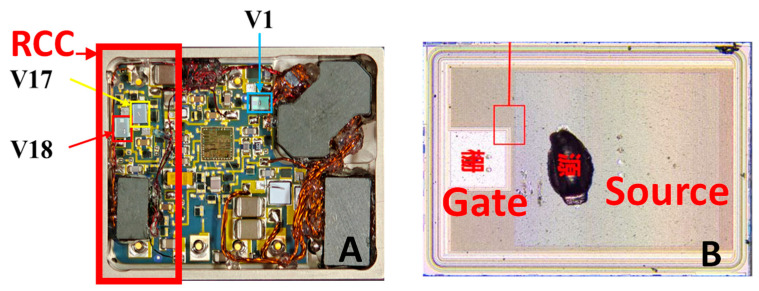
(**A**). Layout diagram of VDMOS; (**B**).VDMOS OM top view.

The irradiation experiments were carried out at the Lanzhou Radiation Technology Development Center of the China Nuclear 404 Institute using a ^60^Co γ-ray source. Six radiation-hardened DC/DC converter modules were selected as test samples. The internal structure of the converter is shown in Figure 2. The VDMOS devices (V17 and V18) used in the module were radiation-hardened N-channel MOSFET bare dies with the model number XXXX67124, fabricated by the No. 58 Research Institute of CETC. All VDMOS devices were manufactured from the same wafer lot, and their key electrical parameters are summarized in Table 1. The irradiation conditions and grouping scheme are summarized in Table 2.

During the experiment, the six converter modules were divided into three groups (Groups A, B, and C), with two modules in each group. The samples were irradiated at three dose rates of 3.89, 8.89, and 13.89 rad(Si)/s, respectively, while the accumulated total dose was fixed at 100 krad(Si) for all groups. Note that these dose rates are under accelerated laboratory conditions; typical on-orbit dose rates are orders of magnitude lower, and a lower dose rate would correspond to a much longer exposure duration for the same total dose. To emulate a cold-redundant (powered-off) state and eliminate floating-node potentials, all external module pins were shorted together; thus, the module (and the embedded VDMOS devices) remained in an unbiased, equipotential shorted state throughout irradiation. Detailed irradiation configurations are listed in Table 2, and the pre-irradiation converter data are summarized in Table 3. After irradiation, the DC/DC converters were electrically characterized, and the transfer characteristics of the VDMOS devices were measured using a Keithley (Solon, OH, USA) 4200A-SCS semiconductor parameter analyzer.

## 4. Results and Discussion

### 4.1. System-Level Test Results

Table 4 summarizes the variations of the electrical parameters of the DC/DC converter after total ionizing dose (TID) irradiation under cold redundancy conditions.

As indicated in Table 4, six DC/DC converter modules were subjected to a total ionizing dose of 100 krad(Si) under cold redundancy conditions and were subsequently powered on for electrical characterization. Compared with the pre-irradiation data in Table 3, overall, all converters remained functional after irradiation, and their key electrical parameters exhibited no significant deviation from the pre-irradiation values. A slight reduction in conversion efficiency was observed, which can be attributed to radiation-induced degradation in semiconductor devices. For the VDMOS transistors, TID exposure leads to an increase in on-state resistance and a negative shift in threshold voltage, resulting in elevated conduction and switching losses. To further clarify the impact of irradiation on the auxiliary bias network [3,4], the RCC supply circuit was characterized separately, as discussed in the following subsection.

**Figure 3:**
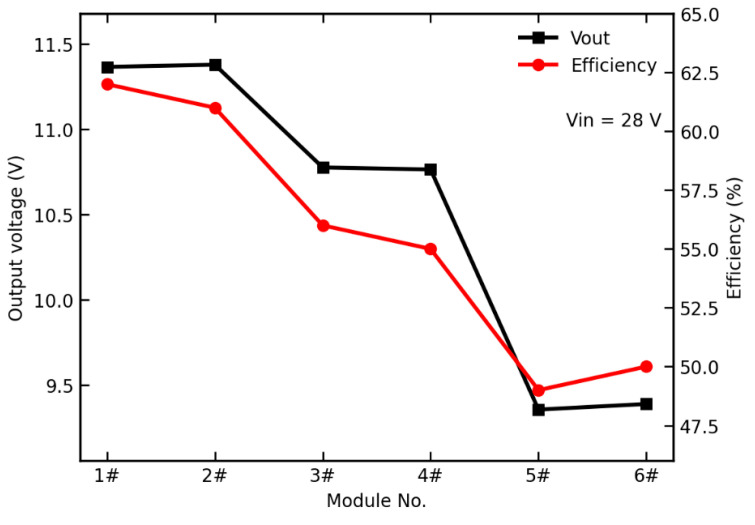
Test data of the RCC circuit after irradiation.

Table 5 and Figure 3 show the measured output characteristics of the RCC auxiliary supply circuit after irradiation. For all six DC/DC converter modules, the RCC output voltage exhibits a noticeable reduction after TID exposure, although the supply voltage remains above the minimum requirement of 8.7 V for proper operation of the PWM controller. Nevertheless, the VDMOS devices have already experienced measurable performance degradation. Under higher dose-rate conditions or at larger accumulated doses, the RCC output voltage is expected to further decrease and may eventually fall below the operating threshold of the PWM controller, leading to startup or functional failure. Therefore, it is necessary to analyze the degradation trends of the VDMOS devices and to propose appropriate radiation-hardening measures. In the following, the underlying reasons for the different RCC output voltage degradations observed at the three dose rates are investigated, with particular emphasis on the radiation-induced performance degradation of the critical VDMOS switching transistors (V17 and V18).

### 4.2. Device-Level Test Results

#### 4.2.1. Comparison Between V17 and V18

**Figure 4:**
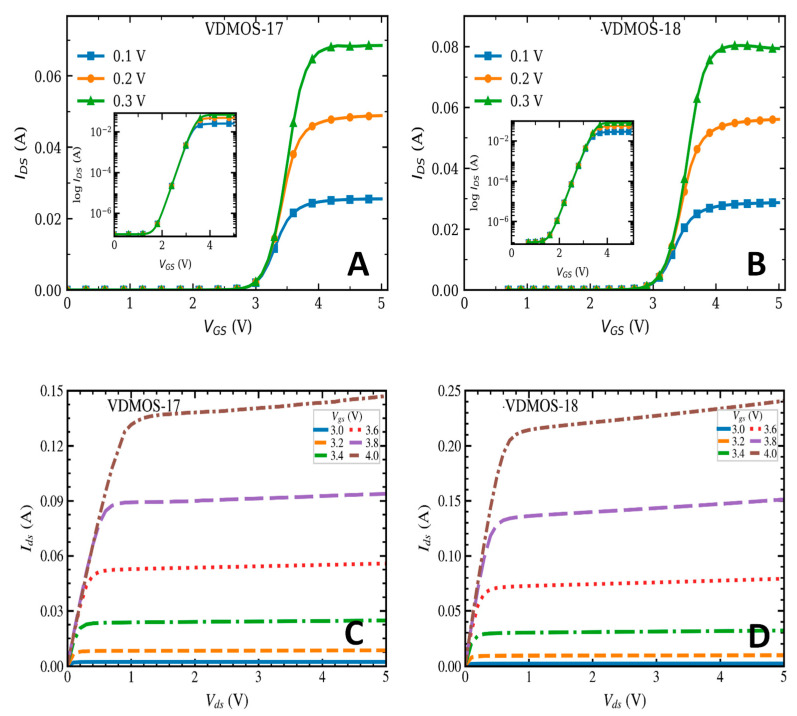
Electrical characteristics of the VDMOS devices; (**A**) Transfer characteristics of V17; (**B**) transfer characteristics of V18; (**C**) output characteristics of V17; (**D**) output characteristics of V18.

The VDMOS devices V17 and V18 in all six DC/DC converter modules were characterized, and similar degradation trends were observed among the samples. Taking sample 2# from Group A as a representative case, Figure 4 shows the measured transfer and output characteristics of V17 and V18 after total ionizing dose irradiation.

Under cold-redundant (unpowered) irradiation at the same dose rate, devices V17 and V18 exhibit highly consistent degradation trends. The dose-dependent evolutions of key parameters, including threshold voltage shift (Δ*V_th_*) and drain leakage current (*I_ds_*), show nearly overlapping curves, indicating similar total-ionizing-dose sensitivity in terms of fabrication process, packaging, and charge-accumulation response. Although the VDMOS devices are irradiated while integrated within the DC/DC converter, no direct electron transport occurs between devices during cold-redundant irradiation. In particular, despite the circuit-level interconnection between V17 and V18 in the present design, the devices remain electrically unbiased and effectively isolated throughout irradiation, with no electric-field-assisted injection or bias-dependent charging effects involved. Consequently, the observed degradation is primarily dominated by ionization-induced buildup of fixed oxide charge and interface-state generation [13,14,15,16,17,18], with both the direction and magnitude of the parameter shifts governed by the intrinsic device properties, thereby enhancing the consistency of degradation behavior between the two devices. In the following, the influence of dose rate on TID-induced degradation is further investigated, with V18 selected as a representative device for comparison.

#### 4.2.2. Comparison of V18 in the DC/DC Converter Under Different Dose Conditions

**Figure 5:**
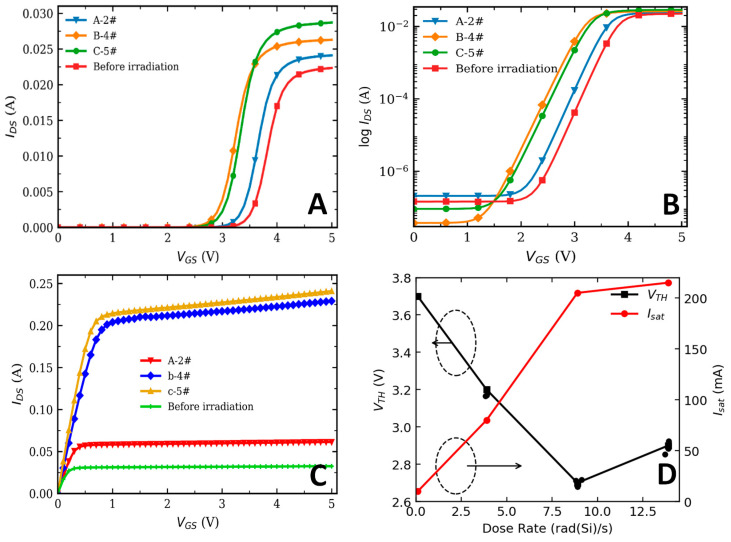
(**A**) Comparison of transfer characteristics of V18 under different dose-rate conditions; (**B**) subthreshold characteristics; (**C**) output characteristics; and (**D**) dependence of threshold voltage and drain saturation current on dose rate.

For the DC/DC converters irradiated under three different dose-rate conditions, one representative sample exhibiting the most severe degradation of the RCC circuit was selected from each group for detailed analysis, namely sample 2# from Group A, 4# from Group B, and 5# from Group C. The experimental results are shown in Figure 5. For each dose-rate condition, two converter modules were irradiated and characterized, and two VDMOS chips were measured from each module (n = 4 devices per dose rate).

As shown in Figure 5A, the transfer characteristics of the VDMOS devices exhibit a clear negative shift in threshold voltage after irradiation. For the A-2# module irradiated at a dose rate of 3.89 rad (Si)/s, the threshold voltage decreases from 3.7 V before irradiation to approximately 3.2 V after an accumulated dose of 100 krad(Si), corresponding to a threshold voltage shift Δ*V_th_* of 0.5 V. For the B-4# module irradiated at 8.89 rad (Si)/s to the same total dose, the threshold voltage decreases to approximately 2.7 V, yielding a larger Δ*V_th_* of about 1.0 V. In contrast, for the C-5# module irradiated at the higher dose rate of 13.89 rad (Si)/s, the threshold voltage decreases to about 2.9 V, corresponding to a Δ*V_th_* of approximately 0.8 V. The comparison of threshold voltage shifts under different dose-rate conditions is summarized in Figure 5D.

The results indicate that, under cold-redundant conditions, the threshold voltage of the VDMOS devices shifts negatively for all dose rates; however, the magnitude of the shift is strongly dose-rate-dependent. Notably, the shift at 13.89 rad(Si)/s is smaller than that observed at 8.89 rad (Si)/s. Under unbiased irradiation, the net trapped-charge buildup is governed by the competition among hole trapping, electron–hole recombination, and time-dependent relaxation/annealing during exposure. For a fixed total dose *D_tot_*, the exposure time scales as t = *D_tot_*/*Ḋ*. In our experiment (*D_tot_* = 100 krad(Si)), the corresponding exposure times were approximately 7.1 h (3.89 rad(Si)/s), 3.1 h (8.89 rad(Si)/s), and 2.0 h (13.89 rad(Si)/s). At a lower dose rate, the much longer irradiation time allows more in-situ relaxation/annealing of radiation-induced charge and defects, partially compensating the net trapped-charge buildup. At higher-dose rate, enhanced carrier recombination reduces the effective hole yield, and prompt recovery processes further suppress the net trapped charge. Consequently, the competition between trapping, recombination, and time-dependent recovery can naturally lead to a maximum net oxide-charge effect at an intermediate dose rate, providing a physically grounded explanation for why 8.89 rad(Si)/s appears as the worst-case condition in Figure 5D under cold-redundant irradiation.

Figure 5B shows the subthreshold characteristics of the VDMOS devices. The subthreshold swing (SS) is related to the interface-state density (*D_it_*) according to the following relationship [9,21]:

(1) $$SS=(\frac{kT}{q})ln10(1+\frac{qD_{it}}{C_{ox}})$$

where$\;C_{ox}\;$ is the gate oxide capacitance, k  is the Bolzmann constant, and T  is the absolute temperature. As can be extracted from the curves, the subthreshold swings of the four curves are nearly identical, indicating that low-dose-rate of total ionizing dose irradiation does not significantly increase the Si/SiO_2_ interface-state density $D_{it}$ nor does it markedly alter the depletion capacitance $C_{d}$. In other words, the irradiation-induced degradation is mainly associated with oxide-trapped charge $Q_{ox}$, rather than interface-state generation [22]. The formation of interface states is generally correlated with irradiation time and accumulated dose level [23], and a total dose of 100 krad(Si) may be insufficient to induce a noticeable increase in $D_{it}$.

Figure 5C illustrates the output characteristics of the VDMOS devices, namely the relationship between the drain current (*I_ds_*) and the drain voltage (*V_ds_*). After cold-redundant total ionizing dose irradiation, no obvious change in the output characteristics is observed at a dose rate of 3.89 rad (Si)/s. When the dose rate increases to 8.89 rad (Si)/s, the *I_DS_*–*V_DS_* curves shift upward significantly, whereas at the higher dose rate of 13.89 rad (Si)/s, the curves tend to stabilize and show reduced sensitivity to further dose-rate increase.

By extracting the on-state resistance from the output characteristics, it is found that sample B-4# exhibits the smallest on-resistance, followed by C-5#, while A-2# shows the largest on-resistance. This trend is opposite to the commonly reported increase in on-resistance under biased irradiation conditions. Equation (2) represents the classical expression of the on-state resistance [21].

(2) $$R_{on}=R_{ch}+R_{acc}+R_{JFET}+R_{D}+R_{sub}$$

where $R_{ch}$ is the channel resistance, $R_{acc}$ is the accumulation-layer resistance, $R_{JFET}$ is the JFET-region resistance, $R_{D}$ is the drift-region resistance, and $R_{sub}$ is the substrate resistance. Under cold-redundant conditions, the device operates in an unbiased or weakly biased state. Total ionizing dose irradiation mainly induces charge trapping in the gate oxide and generates radiation-induced defects at the Si/SiO_2_ interface. These effects directly modify the electrostatics of the gate-controlled channel, leading to threshold voltage shift and carrier mobility degradation, which predominantly affect the channel resistance $R_{ch}$. In contrast, the drift region resistance, JFET region resistance, and substrate resistance are primarily determined by device geometry and doping profiles and are therefore much less sensitive to total ionizing dose effects. As a result, under cold-redundant irradiation conditions, the observed variation in the on-state resistance of vertical power MOSFETs is mainly attributed to the degradation of the channel resistance $R_{ch}$, while the contributions from other resistance components can be neglected [9,22,24]. Equation (3) represents the fundamental physical relationship of the MOSFET channel resistance [25]:

(3) $$R_{ch}\propto \frac{1}{\mu (V_{gs}-V_{th})}$$

when $V_{GS}=4\;V$, a radiation-induced reduction in the threshold voltage $V_{th}$ results in an increased gate overdrive $\left(V_{GS}-V_{th}\right)$, thereby leading to a decrease in the channel resistance $R_{ch}$ and a corresponding reduction in the overall on-resistance $R_{on}$.

The drain saturation current $I_{DS,\mathrm{sat}}$ was extracted from the output characteristics. At $V_{GS}=0.1\;V$, the extracted $I_{DS,\mathrm{sat}}$ under different dose-rate conditions is shown in Figure 5D. For each dose-rate condition, two converter modules were irradiated and characterized, and two VDMOS chips were measured from each module (i.e., n=4 devices per dose rate). All chips were fabricated in the same production lot, providing high part-to-part consistency. Therefore, in Figure 5D, the four scattered points around each $V_{th}$ marker correspond to the individually extracted $V_{th}$ values of the four chips (two chips per module, two modules per condition), illustrating the device-to-device dispersion under identical irradiation and measurement procedures. The square markers connected by lines represent the mean trend of $V_{th}$ versus dose rate. It can also be observed that, for dose rates of 3.89, 8.89, and 13.89 rad(Si)/s, $I_{DS,\mathrm{sat}}$ increases by factors of 2.1, 8.0, and 8.48, respectively. When the dose rate exceeds 8.89 rad(Si)/s, $I_{DS,\mathrm{sat}}$ tends to saturate and shows little further increase.

**Figure 6:**
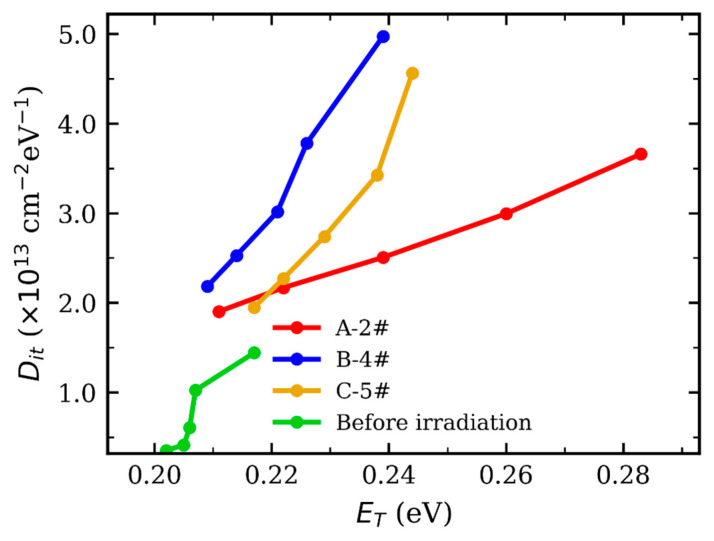
Relationship between the trap energy level ($E_{T}$) and the interface trap density ($D_{it}$).

SS reflects an effective $D_{it}$ contribution and may be insensitive when *N_ot_* dominates; DCIV is energy-resolved and therefore reveals $D_{it}(E)$ differences. Using the Direct-Current Current–Voltage (DCIV) technique [23], the relationship between the trap energy level $E_{T}$ and the interface trap density $D_{it}$ was extracted, as shown in Figure 6. The tests were performed at 300 K, and the DCIV sweep was conducted in a stepwise manner with a 20 ms integration/measurement time per point. For the same total ionizing dose, the low dose-rate condition of 3.89 rad/s results in the most pronounced increase in $D_{it}$. This behavior suggests that defects generated under low dose-rate irradiation are characterized by slower relaxation and annealing kinetics, leading to a more efficient accumulation of interface traps.

## 5. Radiation Hardening Measures

### 5.1. RCC Auxiliary Power Supply and Start-Up Path (Highest Priority)

The nominal 8.7-V threshold should be treated as a design margin rather than a functional operating point. The auxiliary winding, rectification and filtering network, start-up resistor, and VCC energy-storage capacitor should be re-designed based on the worst-case post-irradiation degradation of the RCC output, ensuring that the VCC voltage remains sufficiently higher than both the PWM supply requirement and the UVLO threshold. This prevents operation near critical thresholds, which may otherwise lead to start-up delay or unstable restart behavior after irradiation.

### 5.2. Introduction of UVLO Hysteresis with Controlled Retry/Soft-Start

To mitigate radiation-induced VCC degradation, a UVLO scheme with sufficient hysteresis should be employed to avoid oscillation near the turn-on threshold. In addition, controlled retry or soft-start mechanisms can be introduced to limit start-up stress and abnormal current during repeated restart attempts, thereby improving the re-powering success rate under cold-redundant conditions.

### 5.3. VDMOS (V17/V18) and Power-Stage Hardening Against False Turn-On

A deterministic gate-off bias design should be implemented by adding gate–source pull-down paths and appropriate VGS clamping or limiting circuits. This is particularly important to suppress unintended turn-on caused by radiation-induced threshold voltage reduction combined with transient coupling. Experimental results show that at very low gate bias (e.g., $V_{GS}=0.1\;V$), the drain saturation current can increase by nearly one order of magnitude, making near-off-state conduction a critical risk during cold-redundant restart.

### 5.4. Enhanced Cold-Redundancy Strategy for Reliable Restart

The cold-redundancy strategy should be upgraded from a purely powered-off mode to a periodic bias-refresh and health-monitoring scheme. Short-duration power-up or controlled biasing can be applied periodically, while key indicators such as start-up time, no-load current, and VCC build-up margin are monitored to reveal degradation before functional failure. In addition, input isolation optimization and local shielding resources should be preferentially allocated to the RCC and VDMOS regions, as degradation in this control chain directly determines restart capability even when the main output stage remains functional.

## 6. Conclusions

Under 100 krad (Si) total ionizing dose irradiation in cold-redundant conditions, the optimized RCC isolated DC/DC converter maintains stable main-output voltage and efficiency without evident functional degradation. However, the auxiliary power supply path exhibits significantly higher radiation sensitivity. After irradiation, both the RCC auxiliary supply voltage and efficiency degrade noticeably, resulting in a substantial reduction in the controller supply margin and becoming the primary bottleneck limiting restart reliability under cold-redundant operation. Device-level characterization reveals that the threshold voltage of the critical VDMOS devices experiences a negative shift of approximately 0.5–1.0 V with a clear dose-rate dependence, while the subthreshold swing remains nearly unchanged, indicating that the degradation is dominated by oxide-trapped charge accumulation. Meanwhile, the drain current in the low gate-voltage region increases markedly, which may introduce unintended conduction and stress amplification during turn-off and start-up transients, thereby propagating device-level degradation into system-level risks. Consequently, radiation-hardened design of cold-redundant DC/DC converters should prioritize the auxiliary power supply and start-up margin of the RCC controller, in conjunction with reinforced VDMOS turn-off control and supply protection measures, to enhance long-term restart capability and reliability in high-TID environments.

## Figures and Tables

**Table 1:** Electrical Characteristics of the VDMOS Devices.

Parameters	Test Conditions	Minimum Value	Maximum Value
*V*_(BR)DSS_	*V*_Gs_ = 0 V, *I*_D_ = 1 mA	150 V	-
*V*_GS(th)_	*V*_Ds_ = *V*_Gs,_ *I*_D_ = 1 mA	1.5 V	5 V
*I*_GSSF_	*V*_GS_ = 20 V, *V*_DS_ = 0 V	−200 nA	200 nA
*I*_GSSR_	*V*_GS_ = 20 V, *V*_DS_ = 0 V	-	-
*I*_DSS_	*V*_Gs_ = 0 V, *V*_Ds_ = 80 V	150 V	100 μA
*R*_DS(ON)_	*V*_Gs_ = 12 V, *I*_D_ = 5 A	1.5 V	460 mΩ

**Table 2:** Test Conditions and Ratings.

DC/DC Converter Model	Module No.		Dose Rate (rad(Si)/s)	Total Dose Krad(Si)	Power Module Operating Condition
XXXX-(20-50)-12-15/SP	A	1#	3.89	100	Cold Backup
		2#			Cold Backup
	B	3#	8.89	100	Cold Backup
		4#			Cold Backup
	C	5#	13.89	100	Cold Backup
		6#			Cold Backup

**Table 3:** Electrical parameters of the DC-DC Converter before irradiation.

Module No.	Input Voltage (V)	Output Voltage (V)	Voltage Regulation (mV)	Load Regulation (mV)	Efficiency
1#	28	12.023	26	21	78.9%
2#	28	12.022	28	22	78.8%
3#	28	12.031	27	27	78.8%
4#	28	12.046	25	23	78.6%
5#	28	12.014	21	20	78.9%
6#	28	12.024	24	19	78.9%

**Table 4:** Electrical parameters of the DC-DC Converter after irradiation.

Module No.	Input Voltage (V)	Output Voltage (V)	Voltage Regulation (mV)	Load Regulation (mV)	Efficiency
1#	28	12.034	28	34	78.7%
2#	28	12.029	26	30	78.6%
3#	28	12.038	32	31	78.5%
4#	28	12.035	34	45	78.3%
5#	28	12.034	52	58	78.2%
6#	28	12.039	41	44	78.3%

**Table 5:** Electrical parameters of the RCC after irradiation.

Module No.	Input Voltage (V)	Output Voltage (V)	Load Regulation (mV)	Efficiency
1#	28	11.365	132	62%
2#	28	11.378	141	61%
3#	28	10.775	213	56%
4#	28	10.763	221	55%
5#	28	9.354	452	49%
6#	28	9.387	443	50%

## Data Availability

The original contributions presented in this study are included in the article. Further inquiries can be directed to the corresponding author.
